# Comparative outcomes of microsurgical dorsal root entry zone lesioning (DREZotomy) for intractable neuropathic pain in spinal cord and cauda equina injuries

**DOI:** 10.1007/s10143-024-03136-y

**Published:** 2025-01-02

**Authors:** Bunpot Sitthinamsuwan, Tanawat Ounahachok, Sawanee Pumseenil, Sarun Nunta-aree

**Affiliations:** 1 Division of Neurosurgery, Department of Surgery, Faculty of Medicine Siriraj Hospital, Mahidol University, 2 Wang Lang Road, Bangkok Noi, 10700 Bangkok, Thailand; 2 Department of Surgery, Panyananthaphikkhu Chonprathan Medical Center, Srinakharinwirot University, Nonthaburi, Thailand; 3 Neurosurgical Unit, Division of Perioperative Nursing, Department of Nursing, Faculty of Medicine Siriraj Hospital, Mahidol University, Bangkok, Thailand

**Keywords:** Cauda equina injury, Dorsal root entry zone lesioning, DREZotomy, Neuropathic pain, Spinal cord injury, Surgical outcome

## Abstract

Treatment of neuropathic pain in patients with spinal cord injury (SCI) and cauda equina injury (CEI) remains challenging. Dorsal root entry zone lesioning (DREZL) or DREZotomy is a viable surgical option for refractory cases. This study aimed to compare DREZL surgical outcomes between patients with SCI and those with CEI and to identify predictors of postoperative pain relief. We retrospectively analyzed 12 patients (6 with SCI and 6 with CEI) with intractable neuropathic pain who underwent DREZL. The data collected were demographic characteristics, pain distribution, and outcomes assessed by numeric pain rating scores. Variables and percentages of pain improvement at 1 year and long-term were statistically compared between the SCI and CEI groups. The demographic characteristics and percentage of patients who experienced pain improvement at 1 year postoperatively did not differ between the groups. Compared with the SCI group, the CEI group presented significantly better long-term pain reduction (p = 0.020) and favorable operative outcomes (p = 0.015). Patients with border zone pain had significantly better long-term pain relief and outcomes than did those with diffuse pain (p = 0.008 and p = 0.010, respectively). Recurrent pain after DREZL occurred in the SCI group but not in the CEI group. DREZL provided superior pain relief in patients with CEI. The presence of border zone pain predicted favorable outcomes. CEI patients or SCI patients with border zone pain are good surgical candidates for DREZL, whereas SCI patients with below-injury diffuse pain are poor candidates.

## Introduction

Neuropathic pain is a major consequence of nervous system injuries or diseases [1, 2]. These injuries can occur at various sites, including the peripheral nerves, nerve plexuses, nerve roots, spinal cord, and brain [3–6]. Among them, spinal cord injury (SCI) and cauda equina injury (CEI) are common neurological insults associated with neuropathic pain [7–11].

Neuropathic pain following SCI and CEI is generally managed with various medical therapies and nonoperative modalities [12–16]. However, many patients develop severe neuropathic pain refractory to conventional treatments [17, 18]. In such cases, neurosurgical procedures play a major role in treating intractable neuropathic pain [19–26]. Dorsal root entry zone lesioning (DREZL) or DREZotomy is an effective option for pain relief in various pain conditions, including neuropathic pain from brachial plexus injury, SCI, CEI, herpes infection, and limb amputation [27–32]. Additionally, DREZL is effective in relieving severe limb spasticity and dystonia [33–39].

Previous studies have reported the outcomes of DREZL for neuropathic pain caused by SCI and CEI [40, 41]. However, no study has statistically compared the surgical outcomes of DREZL between patients with SCI and those with CEI. Therefore, we conducted this study to compare the surgical outcomes of DREZL between these two groups and to investigate predictors of favorable operative outcomes.

## Materials and methods

### Patient population

This retrospective study included patients with refractory neuropathic pain resulting from either SCI or CEI who underwent DREZL at our medical institute between January 2013 and December 2023. All patients had neuropathic pain unresponsive to medical treatment, rehabilitation, physical therapy, and interventional pain management prior to consideration for DREZL. Patients with concomitant SCI and CEI were excluded. The recruited patients were classified into two groups: those with neuropathic pain caused by SCI (SCI group) and those with neuropathic pain caused by CEI (CEI group).

### Surgical considerations and operative techniques

Regarding our treatment of intractable pain, DREZL was considered in SCI or CEI patients who had completely or partially paralyzed lower extremities without hope of regaining motor function (non-functional lower extremities). This operation was not utilized in patients with significant residual motor strength of the lower limbs or in patients who could independently stand or walk (functional lower extremities). In patients with refractory pain of functional limbs, spinal cord stimulation was utilized as a primary neurosurgical treatment.

Preoperative identification of the distribution of neuropathic pain and the corresponding dermatomes was routinely performed. On the basis of this mapping, the targeted spinal cord segments for DREZL were determined. Spinal magnetic resonance imaging was used to identify the degree of injury and assess damage to neural structure. Additional three-dimensional spinal computed tomography images were obtained from patients with metallic artifacts on imaging resulting from spinal fixation hardware. The operative techniques for DREZL are described below.

1. Anesthesia and positioning.

Surgery was performed under general anesthesia without neuromuscular blockade. An endotracheal tube was used for airway protection and ventilation. Muscle relaxants were avoided to permit intraoperative neurophysiological testing. Patients were positioned prone, as in standard spinal surgery.

2. Intraoperative neurophysiological monitoring and identification of spinal levels.

The targeted spinal cord segments were anatomically and electrophysiologically identified during DREZL procedures. Intraoperative electromyography (EMG) and electrical nerve stimulation were routinely employed. EMG needles were inserted into key lower limb muscle groups: the quadriceps femoris, tibialis anterior, medial gastrocnemius, and medial hamstring muscles. The EMG was used to record compound muscle action potentials (CMAPs) evoked by electrical nerve stimulation. Bilateral monitoring was performed even when DREZL was conducted unilaterally. The vertebral levels used for laminectomy to access the targeted dorsal root entry zone (DREZ) were labeled via intraoperative fluoroscopy.

3. Preparation of operative field.

After demarcating the targeted vertebral levels for laminectomy, a longitudinal midline incision was marked to encompass the uppermost and lowermost levels. The operative field was prepared via standard sterile techniques. Both lower extremities were covered with a large sterile transparent plastic sheet to allow visual inspection of muscle contractions during activation of the spinal motor nerve roots.

4. Operative exposure.

A midline incision was made to access the spinous processes and laminae of the targeted spinal levels. These structures were optimally removed to expose the spinal epidural space. The dura mater and arachnoid mater were then opened rostrally and caudally to adequately expose the targeted spinal cord segments for the DREZL procedure.

5. Localization of targeted spinal cord segments via electrical nerve stimulation and intraoperative EMG.

Each spinal motor nerve root within the operative field was stimulated with 0.5 mA of electrical current. Concurrently, contractions of the lower limb muscle groups beneath the sterile plastic sheet and the appearance of CMAPs on EMG were observed. The patterns of muscle contraction and CMAP occurrence in specific lower limb muscles facilitated the identification of the stimulated spinal motor nerve root. For example, activation of the L2 or L3 spinal motor nerve root produced vigorous contraction of the quadriceps femoris (ipsilateral knee extension) and concurrent CMAPs on EMG. Similarly, stimulation of the L4 or L5 spinal motor nerve root resulted in ipsilateral ankle dorsiflexion due to contraction of the tibialis anterior muscle, with simultaneous CMAP detection via EMG.

After the stimulated spinal motor nerve root was identified, it was traced proximally to its corresponding spinal cord segment, thereby localizing that segment. This technique enables the precise identification of the uppermost and lowermost targeted spinal cord segments for DREZL. This method has proven to be relatively accurate for localizing consecutive spinal cord segments.

However, in patients with CEI and multiple types of spinal nerve root dysfunction, this localization technique is limited because stimulation of severely injured spinal motor nerve roots results in an absence of motor responses and CMAPs on EMGs. In such cases, we attempted to stimulate the contralateral parallel spinal motor nerve root. If activation of the contralateral nerve root elicited physical and electrophysiological responses, the targeted spinal cord segments can still be localized via the technique described above. Conversely, if there was no response upon stimulation of the bilateral spinal motor nerve roots, localization of the targeted spinal cord segments for DREZL had to rely solely on anatomical landmarks at the vertebral level.

We routinely utilize this electrophysiological method for localizing spinal cord segments during lesioning procedures, such as DREZL for lower limb pain or spasticity [38, 42], Bischof II myelotomy for spastic paraplegia [43], and cordotomy for lower limb cancer pain. On the basis of our experience, the relationship between spinal cord segments and vertebral levels in most patients—with the conus medullaris ending at the junction between the L1 and L2 vertebral levels—can be summarized as follows:

- **L1 spinal cord segment**: approximately lower part of the T9 vertebra to upper part of the T10 vertebra.
- **L2 spinal cord segment**: approximately upper to lower part of the T10 vertebra.
- **L3 spinal cord segment**: approximately lower part of T10 to upper part of T11 vertebral levels.
- **L4 spinal cord segment**: approximately upper to lower part of the T11 vertebra.
- **L5 spinal cord segment**: approximately lower part of T11 to upper part of T12 vertebral levels.
- **S1 spinal cord segment**: approximately upper to lower part of the T12 vertebra.
- **S2 spinal cord segment**: approximately lower part of T12 to upper part of L1 vertebral levels.

This relationship may assist in the anatomical localization of spinal cord segments using vertebral levels, especially in cases of bilateral severe CEI or when electrical nerve stimulators or intraoperative EMGs are unavailable during surgery.

**Fig. 1:**
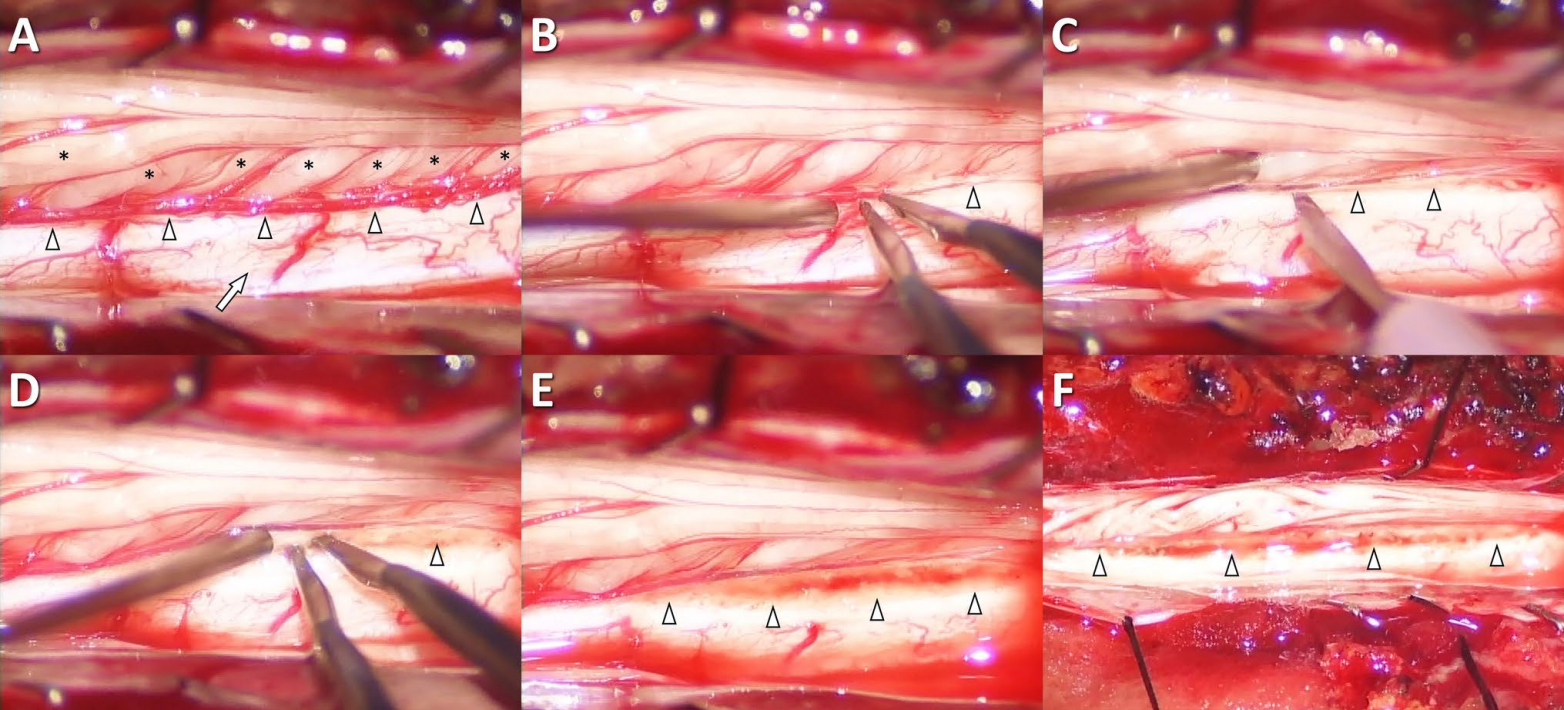
Intraoperative procedures of DREZL for pain relief in a patient with neuropathic pain of the non-functional lower extremities. **(A)** exposure of the posterolateral surface of the spinal cord (arrow) and DREZ (arrowheads) after posterior reflection of the dorsal spinal nerve roots (asterisks); **(B)** coagulation of small arteries covering the DREZ (arrowhead); **(C)** incision along the DREZ (arrowheads) using an ophthalmic microsurgical blade; **(D)** microsurgical coagulation of the DREZ (arrowhead) using fine-tip bipolar coagulation forceps with adequate depth and correct trajectory; **(E)** exposure of the dorsolateral sulcus (arrowheads) of the spinal cord after DREZL; **(F)** complete lesioning along the DREZ (arrowheads) of the targeted spinal cord segments. DREZ, dorsal root entry zone; DREZL, dorsal root entry zone lesioning

6. Procedure for lesioning microsurgical DREZL **(**Fig. 1**)**.

After the uppermost and lowermost spinal cord segments designated for lesioning were marked, the dorsal (sensory) spinal nerve roots were reflected posteriorly to expose the DREZ. The DREZ is located just anterior to where the dorsal spinal nerve roots attach to the spinal cord. An ophthalmic microsurgical knife was used to open the pia mater of the spinal cord. An incision was made along the targeted DREZ to access the dorsolateral sulcus.

We performed microsurgical DREZL using the operative technique proposed by Sindou et al. [28, 31, 33, 44–46]. In SCI patients, we performed DREZL on the spinal cord segments corresponding to the painful area, including one additional segment above and below the targeted segment. In contrast, we performed DREZL only on the spinal cord segments corresponding to the painful area in CEI patients.

Lesioning of the DREZ was conducted via bipolar coagulation with 0.2 mm-tip Caspar-style forceps (Aesculap). The angle of coagulation was 45 degrees relative to the spinal cord, and the depth of lesioning was 3–4 mm from the spinal cord surface. Complete coagulation was achieved along the DREZ of the targeted spinal cord segments. In patients with bilateral symmetrical pain, an identical lesioning procedure was performed on the contralateral side.

7. Closure of the dura mater and incision.

The dura mater was sutured tightly to prevent cerebrospinal fluid leakage. A vacuum drain catheter was placed in the epidural space in most patients. The paraspinal muscles, lumbar fascia, subcutaneous tissue, and skin were sutured appropriately from deep to superficial layers.

### Data collection

We collected the demographic characteristics and operative outcomes of patients who underwent DREZL for analysis. The demographic data were sex, age, etiology of neuropathic pain (SCI or CEI), and mechanism of injury (closed or gunshot injury). We also recorded the duration of preoperative pain, distribution of neuropathic pain (diffuse or border zone pain), pain dermatomes, spinal cord segments where DREZL was performed, and duration of postoperative follow-up. The operative outcome data comprised baseline numeric pain rating scores (NPRSs) before DREZL, NPRS at 1 year postoperatively, NPRS at the last follow-up (long-term), and any surgical complications. “Diffuse pain” was defined as pain below the level of the SCI that spread downward to the bilateral lower extremities and perineal area. “Border zone pain” was segmental pain—a band of pain—with well-demarcated upper and lower borders.

### Outcome assessment

Operative outcomes were assessed by calculating the percentage change in NPRS after surgery compared with the preoperative baseline value. “Pain improvement” was a positive percentage change in postoperative NPRS compared with the preoperative baseline, whereas “pain deterioration” was a negative percentage change. This percentage change was compared between the SCI and CEI groups. Additionally, pain improvement of ≥ 70% after surgery was deemed favorable, whereas improvement of < 70% was classified as unfavorable. The degree of pain improvement after surgery was also compared between the SCI and CEI groups. Operative complications were recorded individually.

### Statistical analysis

The data were analyzed with IBM SPSS Statistics, version 24.0 (IBM Corp, Armonk, NY, USA). Numerical data are presented as means with standard deviations or medians with ranges. Categorical data are reported as numbers with percentages. Comparisons of two independent categorical variables were performed using Fisher’s exact test. An independent t test was used to compare two independent numerical parameters with a normal distribution. The Mann‒Whitney U test was employed for independent numerical variables with skewed distributions. A *p* value of less than 0.05 was considered statistically significant.

## Results

### Demographic characteristics and operative outcomes

After excluding patients with concomitant SCI and CEI, 12 patients were included in the study. The mean age of the patients was 45.2 ± 14.1 years, and the median postoperative follow-up period was 45.5 months (range, 17‒104 months). Among these patients, 6 experienced neuropathic pain caused by SCI (the SCI group), and the remaining 6 experienced neuropathic pain due to CEI (the CEI group).

In the SCI group, patients had injuries at vertebral levels T8 (2 patients), T9 (1 patient), and T12 (3 patients). Diffuse pain was observed in 4 of the 6 SCI patients (66.7%), whereas the remaining 2 SCI patients (33.3%) experienced border zone pain. All the SCI patients (100%) had bilateral pain dermatomes. Following DREZL, 4 of the 6 SCI patients (66.7%) showed immediate improvement in pain of more than 50% compared with the preoperative baseline. However, 2 patients (Patients 1 and 4) developed recurrent neuropathic pain several months after surgery. The recurrent pain in Patient 1 failed to respond to a spinal cord stimulation trial. Patient 4 had previously failed a spinal cord stimulation trial before experiencing recurrent pain after DREZL.

In the CEI group, 4 patients had injuries at the L2 vertebral level, and 2 patients had injuries at the L3 level. All the CEI patients (100%) experienced border zone pain. Three of the 6 CEI patients (50%) presented with unilateral pain dermatomes, whereas the remaining 3 (50%) presented with bilateral pain dermatomes. There was no recurrent pain after surgery in the CEI group.

The demographic characteristics and operative outcomes of all the SCI and CEI patients are summarized in **Table **1.

**Table 1:** Demographic characteristics and surgical outcomes

Patient	Sex/age (years)	Etiology	Lower limb function	Vertebral level of injury	Mechanism of injury	Preop pain duration (months)	Neuropathic pain		Spinal cord segments of DREZL	Preop NPRS	1-year postop NPRS	Long-term postop NPRS	Postop follow-up (months)	Remarks
							Distribution	Pain dermatomes						
1	M 34	SCI	Completely paralyzed	T12	Gunshot	81	Diffuse	Rt T12 and below, Lt L4 and below	Rt T11-S3, Lt L3-S3	10	1	8	89	Recurrent pain at 37 months after DREZL. Recurrent pain was failed to SCS trial
2	M 47	SCI	Completely paralyzed	T8	Closed	31	Diffuse	Bil T10 and below	Bil T9-S3	9	6	7	32	
3	F 55	SCI	Completely paralyzed	T9	Closed	13	Diffuse	Bil T8 and below	Bil T7-S3	10	6	6	23	
4	M 32	SCI^a^	Partially paralyzed	T12	Closed	25	Diffuse	Bil L1 and below	Bil T12-S3	9	2	10	17	Pain was failed to SCS trial before DREZL. Recurrent pain at 11 months after DREZL
5	M 35	SCI	Completely paralyzed	T12	Closed	15	Border zone	Bil L1-L3	Bil T12-L4	10	2	2	53	
6	M 67	SCI	Completely paralyzed	T8	Closed	46	Border zone	Bil T10-T12	Bil T9-L1	10	3	4	38	
7	M 45	CEI	Partially paralyzed	L2	Closed	122	Border zone	Rt L2-L5, Lt S1-S2	Rt L2-L5, Lt S1-S2	8	3	2	104	Transient Lt lower limb weakness after DREZL
8	M 33	CEI	Partially paralyzed	L2	Gunshot	26	Border zone	Bil L1-L3	Bil L1-L3	9	0	0	97	Transient Bil proximal lower limb weakness after DREZL
9	M 45	CEI	Completely paralyzed	L2	Gunshot	66	Border zone	Lt L1-S1	Lt L1-S1	10	0	0	80	
10	M 75	CEI	Partially paralyzed	L2	Closed	338	Border zone	Rt L1-L3	Rt L1-L3	9	1	2	68	Permanent Rt proximal lower limb weakness after DREZL
11	M 33	CEI	Completely paralyzed	L3	Gunshot	31	Border zone	Bil L4-S1	Bil L4-S1	10	2	3	26	
12	F 41	CEI	Completely paralyzed	L3	Closed	14	Border zone	Lt L4-S1	Lt L4-S1	9	1	1	21	

^a^ Incomplete SCI in one patient; the remaining 5 patients had complete SCI

Bil, bilateral; CEI, cauda equina injury; DREZL, dorsal root entry zone lesioning; F, female; L, lumbar; Lt, left; M, male; NPRS, numeric pain rating score; postop, postoperative; preop, preoperative; Rt, right; S, sacral; SCI, spinal cord injury; SCS, spinal cord stimulation; T, thoracic

### Comparison of demographic characteristics and operative outcomes between SCI and CEI groups

The demographic characteristics and operative outcomes of the SCI and CEI groups were compared (**Table **2). No statistically significant differences were found in sex, age, mechanism of injury, preoperative pain duration, pain distribution, laterality of pain dermatomes, symmetry of pain dermatomes, or duration of postoperative follow-up. There was no significant difference in pain improvement (*p* = 0.110) or favorable outcomes (*p* = 0.999) 1 year after surgery compared with the preoperative baseline.

**Table 2:** Comparison of demographic characteristics and surgical outcomes between the spinal cord injury and cauda equina injury groups

				Overall patients (*n* = 12)	SCI vs. CEI groups		
					SCI group (*n* = 6)	CEI group (*n* = 6)	*p* value
Sex, n (%)							1.000
	Male			10 (83.3)	5 (83.3)	5 (83.3)	
	Female			2 (16.7)	1 (16.7)	1 (16.7)	
Age (years), mean ± SD				45.2 ± 14.1	45 ± 14	45.3 ± 15.5	0.970
Mechanism of injury, n (%)							0.545
		Closed injury		8 (66.7)	5 (83.3)	3 (50)	
		Gunshot injury		4 (33.3)	1 (16.7)	3 (50)	
Preoperative pain duration (months), median (range)				31 (13–338)	28 (13–81)	48.5 (14–338)	0.337
Pain distribution, n (%)							0.620
		Diffuse pain^b^		4 (33.3)	4 (66.7)	0 (0)	
		Border zone pain^c^		8 (66.7)	2 (33.3)	6 (100)	
Laterality of pain dermatome, n (%)							0.182
		Right		1 (8.3)	0 (0)	1 (16.7)	
		Left		2 (16.7)	0 (0)	2 (33.3)	
		Bilateral		9 (75)	6 (100)	3 (50)	
Symmetry of pain dermatome^d^, n (%)							0.242
		Symmetrical		7 (58.3)	5 (83.3)	2 (33.3)	
		Aymmetrical		5 (41.7)	1 (16.7)	4 (66.7)	
Percentage change^e^ (%) in 1-year postoperative NPRS compared with preoperative NPRS, median (range)				+ 80 (+ 33.3 to + 100)	+ 73.9 (+ 33.3 to + 90)	+ 88.9 (+ 62.5 to + 100)	0.110
Percentage change^e^ (%) in long-term postoperative NPRS compared with preoperative NPRS, median (range)				+ 72.5 (−11.1 to + 100)	+ 31.1 (−11.1 to + 80)	+ 83.4 (+ 70 to + 100)	0.020^a^
Percentage change^e^ (%) in long-term postoperative NPRS compared with 1-year postoperative NPRS, median (range)				−5 (−88.9 to + 12.5)	−10.6 (−88.9 to 0)	0 (−11.1 to + 12.5)	0.174
1-year postoperative outcome, n (%)							0.999
		Favorable^f^		9 (75)	4 (66.7)	5 (83.3)	
		Unfavorable^g^		3 (25)	2 (33.3)	1 (16.7)	
Long-term postoperative outcome, n (%)							0.015^a^
		Favorable^f^		7 (58.3)	1 (16.7)	6 (83.3)	
		Unfavorable^g^		5 (41.7)	5 (83.3)	0 (16.7)	
Operative complication, n (%)							0.182
			Present	3 (25)	0 (0)	3 (50)	
			Absent	9 (75)	6 (100)	3 (50)	
Duration of postoperative follow-up (months), median (range)				45.5 (17–104)	35 (17–89)	74 (21–104)	0.298

^a^ Indicates a statistically significant difference

^b^ Pain below the level of SCI spreading downward to the bilateral lower extremities and perineal area

^c^ Segmental pain with well-demarcated upper and lower borders of the pain area

^d^ Symmetry of pain dermatomes between the left and right sides

^e^ Percentage change in the numeric pain rating score; the symbol plus (+) refers to pain improvement; the symbol minus (-) refers to pain deterioration; and 0 refers to unchanged pain

^f^ Improvement in the numeric pain rating score ≥ 70% after DREZotomy compared with the preoperative score

^g^ Improvement in the numeric pain rating score < 70% after DREZotomy compared with the preoperative score

CEI, cauda equina injury; n, number of patients; NPRS, numeric pain rating score; SCI, spinal cord injury; SD, standard deviation

However, the CEI group demonstrated significant pain improvement (*p* = 0.020) and favorable outcomes (*p* = 0.015) in the long-term follow-up compared with the preoperative baseline values. No significant difference in pain improvement was observed between the groups when the long-term-follow-up and 1-year-postoperative values were compared (*p* = 0.174).

### Comparison of operative outcomes between pain distribution groups

The operative outcomes of patients with diffuse pain and those with border zone pain were compared (Table 3). There was no statistically significant difference in pain improvement (*p* = 0.174) or favorable outcomes (*p* = 0.491) 1 year after DREZL compared with the preoperative baseline. Patients with border zone pain exhibited significant pain improvement (*p* = 0.008) and favorable outcomes (*p* = 0.010) in the long-term follow-up compared with the preoperative baseline. No significant difference in pain improvement was found between the two pain distribution groups between their long-term follow-up and 1-year postoperative values (*p* = 0.077).

**Table 3:** Comparison of surgical outcomes between patients with diffuse pain and those with border zone pain

		Diffuse pain^b^ (*n* = 4)	Border zone pain^c^ (*n* = 8)	*p* value
Percentage change^d^ (%) in 1-year postoperative NPRS compared with preoperative NPRS, median (range)		+ 58.9 (+ 33.3 to + 90)	+ 84.5 (+ 62.5 to + 100)	0.174
Percentage change^d^ (%) in long-term postoperative NPRS compared with preoperative NPRS, median (range)		+ 21.1 (−11.1 to + 40)	+ 78.9 (+ 60 to + 100)	0.008^a^
Percentage change^d^ (%) in long-term postoperative NPRS compared with 1-year postoperative NPRS, median (range)		−40.6 (−88.9 to 0)	0 (−11.1 to + 12.5)	0.077
1-year postoperative outcome, n (%)				0.491
	Favorable^e^	2 (50)	7 (87.5)	
	Unfavorable^f^	2 (50)	1 (12.5)	
Long-term postoperative outcome, n (%)				0.010^a^
	Favorable^e^	0 (0)	7 (87.5)	
	Unfavorable^f^	4 (100)	1 (12.5)	

^a^ Indicates a statistically significant difference

^b^ Pain below the level of SCI spreading downward to the bilateral lower extremities and perineal area

^c^ Segmental pain with well-demarcated upper and lower borders of the pain area

^d^ Percentage change in the numeric pain rating score; the symbol plus (+) refers to pain improvement; the symbol minus (-) refers to pain deterioration; and 0 refers to unchanged pain

^e^ Improvement in the numeric pain rating score ≥ 70% after DREZotomy compared with the preoperative score

^f^ Improvement in the numeric pain rating score < 70% after DREZotomy compared with the preoperative score

n, number of patients; NPRS, numeric pain rating score

### Complications

Operative complications were encountered exclusively in 3 patients in the CEI group (Patients 7, 8, and 10). Two patients (Patients 7 and 8) experienced transient lower limb weakness, whereas one patient (Patient 10) developed a permanent motor deficit in the lower limb. There was no significant difference in complication rates between the SCI and CEI groups (*p* = 0.182).

## Discussion

Neuropathic pain has been defined as pain resulting from pathology within the nervous system [47]. Its etiologies include traumatic injury to neural structures, nerve compression, infections, channelopathies, and autoimmune disorders [48]. Various mechanisms are associated with the occurrence of neuropathic pain, such as functional alterations in ion channels, immune cell activation, and cellular toxicity [49]. Typically, neuropathic pain is managed successfully with pharmacological treatments, physiotherapy, and pain interventions. However, a subset of patients develops persistent pain that is resistant to conventional treatments. In these cases, neurosurgical procedures have become a viable option [25, 26].

Neuropathic pain following SCI or CEI poses significant challenges for healthcare providers. This pain is often severe, chronic, difficult to treat, and refractory to standard treatment modalities [17, 20, 50, 51]. The precise mechanisms underlying the development of neuropathic pain after SCI or CEI remain incompletely understood [52]. Studies have consistently shown that neuropathic pain is associated with alterations in spinal neuronal function, including loss of inhibition and hyperexcitability of spinal neurons, reactive gliosis, activation of astrocytes and microglia, disruptions in the spinothalamic pathway, and changes in sodium channel expression [52–59]. Additionally, peripheral pain generators and brain structures, such as the thalamus, contribute to the development of neuropathic pain following SCI or nerve root injury [60–64].

Various neurosurgical procedures are employed to treat chronic neuropathic pain. These include neuromodulation techniques such as spinal cord stimulation, intrathecal drug delivery, peripheral nerve stimulation, deep brain stimulation, and neuroablative procedures such as DREZL [65–79]. Among neuroablative techniques, DREZL is effective for treating refractory pain associated with brachial plexus avulsion injury, lumbosacral plexus avulsion injury, SCI, CEI, severe spasticity, and phantom limb pain [27–29, 31–34, 38, 39, 42]. The mechanisms by which DREZL alleviates pain include the interruption of nociceptive fibers at the dorsolateral sulcus, which project to the dorsal horn neurons of the spinal cord, and the inhibition of hyperactive dorsal horn neuron function [28, 33, 44–46, 80].

DREZL has been used for several decades to treat neuropathic pain resulting from SCI and CEI [81]. This procedure has proven effective in relieving neuropathic pain in the majority of patients with SCI or CEI [40, 42, 44, 82–103]. Most patients report improvements in quality of life (general activity, mood, sleep, and enjoyment) following DREZL [100].

In recent years, DREZL has been gradually replaced by neuromodulation therapies, especially spinal cord stimulation and intrathecal drug delivery. However, when neuromodulation devices are unavailable or unaffordable, DREZL remains a crucial option for pain relief in refractory cases. In Thailand, the expense of spinal cord stimulation and intrathecal drug delivery devices is generally not covered by the Universal Coverage Scheme, a government health insurance program. Therefore, we continue to utilize DREZL to treat intractable neuropathic pain in well-selected patients.

In our surgical practice, suitable candidates for DREZL include patients with neuropathic pain who have not regained neurological function, such as SCI or CEI patients with permanent motor or sensory impairments of the extremities. Conversely, spinal cord stimulation is chosen primarily for patients with significant residual motor or sensory function, or for those whose pain distribution overlaps areas of intact sensory function.

In our analysis, patients with SCI were distinctly separated from those with CEI: no patient had concomitant SCI and CEI. We categorized neuropathic pain into diffuse pain and border zone pain. Diffuse pain was described as pain below the level of SCI, radiating downward to the bilateral lower extremities and perianal area. We defined border zone pain as segmental pain with clear upper and lower margins. Four of the 6 patients in the SCI group had diffuse pain, with the other 2 SCI patients experiencing border zone pain. Conversely, all 6 patients in the CEI group experienced border zone pain. However, this difference did not reach statistical significance.

Furthermore, all the SCI patients experienced bilateral pain, whereas only half of the CEI patients developed bilateral lower limb pain. These findings suggest that pain caused by SCI tends to be diffuse and bilateral. Diffuse pain is a major consequence of direct spinal cord damage [47, 63, 64, 104, 105]. The mechanisms underlying diffuse pain remain unclear. After SCI, the portion of the spinal cord above the injury level remains intact. Possible origins of pain include interruption or degeneration of the spinothalamic pathway, occurrence of phantom pain in deafferented body areas, or central mechanisms involving the thalamus and cerebral cortex [106–108].

In contrast, border zone pain may result from spinal nerve root damage, CEI, or intrinsic spinal cord injury [17, 47, 59, 63, 64, 104, 105]. Spinal nerve root injury can occur from the initial trauma or develop later due to vertebral instability or impingement by facet joints or intervertebral discs [109]. Several mechanisms can explain the etiology of border zone pain. First, the loss of inputs to the spinal cord results in altered central connectivity and neuronal activity. Second, damaged nerve roots of the cauda equina may spontaneously generate pain signals. Third, mechanical irritation of the cauda equina and restricted movement of the spinal nerve roots caused by arachnoid adhesions contribute to pain. Fourth, peripheral stimuli may evoke abnormal signals at the site of the axonal injury [47].

Our findings align with these pathophysiological mechanisms underlying the occurrence of neuropathic pain after SCI and CEI.

Regarding operative outcomes, no significant differences were observed in the comparative analysis of one-year surgical outcomes between the SCI and CEI groups. However, during long-term postoperative follow-up, 2 of the 6 patients in the SCI group developed recurrent neuropathic pain. We found that the median percentage of pain improvement and the number of patients with favorable outcomes were significantly greater in the CEI group during the long-term follow-up (*p* = 0.020 and *p* = 0.015, respectively). These results indicate that neuropathic pain caused by CEI responds better to DREZL than does pain caused by SCI. This difference may be attributed to the fact that all individuals in the CEI group had border zone pain, which responds well to the operation. Previous studies have also shown that CEI patients with neuropathic pain have better surgical outcomes with DREZL than do SCI patients with neuropathic pain [40, 41]. However, one study reported effective pain relief with DREZL in SCI patients but not in CEI patients [110].

The notion that neuropathic pain relief with DREZL is more effective in patients with border zone pain than in those with diffuse pain is supported by our study and previous publications [44, 90–92, 96, 101, 111–113]. Our results demonstrated that long-term pain relief in the group with border zone pain was significantly better than that in the group with diffuse pain (*p* = 0.008 for the median percentage of pain improvement and *p* = 0.010 for the number of patients with favorable outcomes). Notably, all 4 SCI patients with diffuse pain experienced unfavorable pain relief during long-term follow-up. These findings suggest that patients with a border zone pattern of neuropathic pain are suitable surgical candidates for DREZL. Conversely, DREZL is not an optimal therapeutic option for diffuse neuropathic pain; neuromodulation or intrathecal drug delivery should be considered instead.

**Fig. 2:**
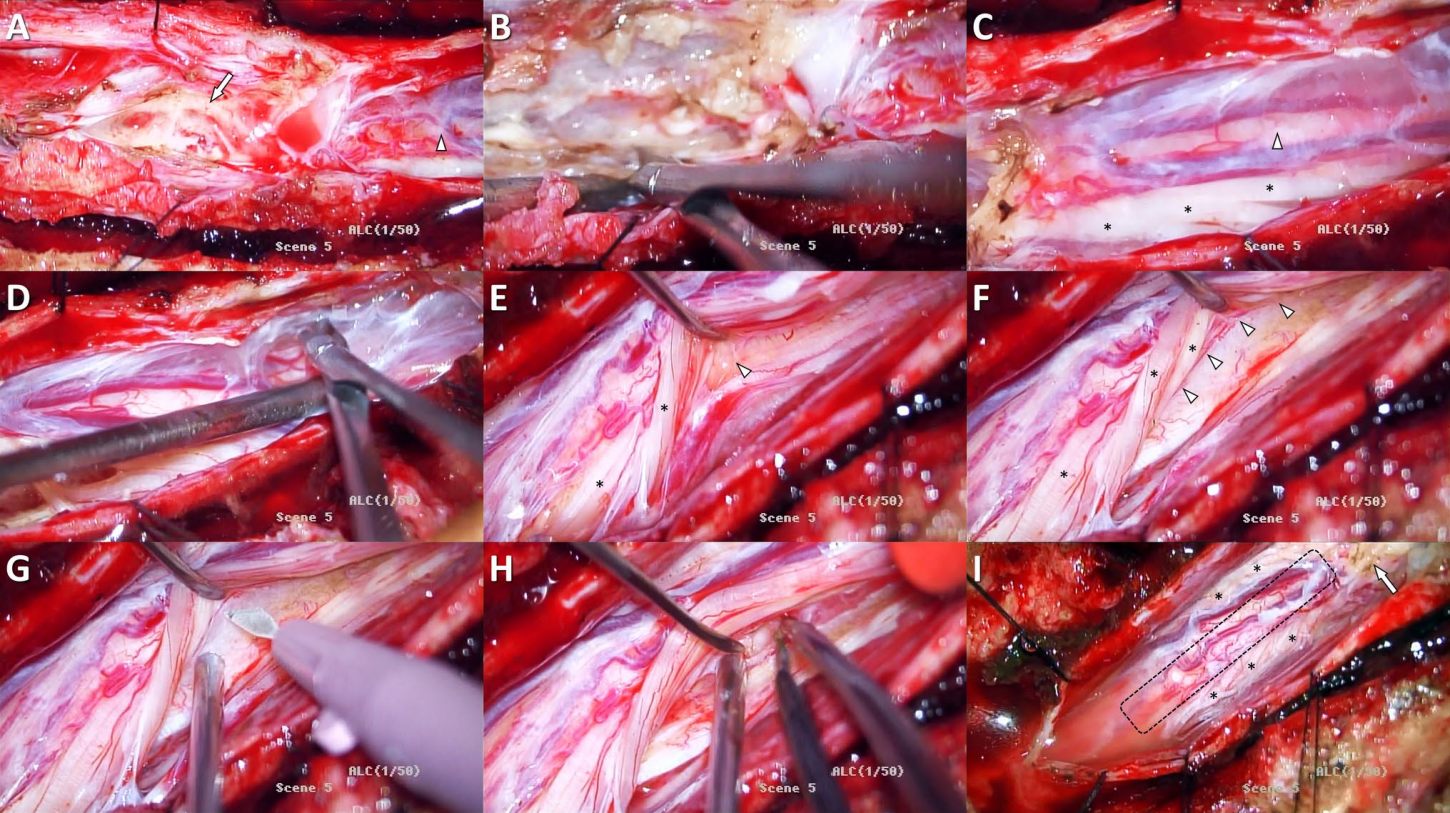
Intraoperative findings and DREZL procedure in a SCI patient with neuropathic pain of the lower limbs. The patient had diffuse pain below the injury level. The targeted spinal cord segments for the lesioning were inferior to the level of extensive spinal cord damage. Arachnoid adhesions and gliotic formation adjacent to the injury site might obscure intraoperative identification of the DREZ. **(A)** the area of SCI (arrow) and intact targeted spinal cord segments for DREZL located below level of the injury (arrowhead); **(B)** excision of gliotic tissues and adhesions around the injured spinal cord; **(C)** the thick arachnoid mater covering the intact spinal cord (arrowhead) and dorsal spinal nerve roots (asterisks) below level of the injury; **(D)** dissection of the arachnoid membranes away from the spinal cord and nerve roots; **(E)** posterior mobilization of the dorsal spinal nerve roots (asterisks) to expose the posterolateral surface of the spinal cord (arrowhead); **(F)** identification of the DREZ (arrowheads) after reflection of the dorsal spinal nerve roots (asterisks); **(G)** incision of the DREZ using an ophthalmic microsurgical blade; **(H)** microsurgical coagulation of the DREZ with appropriate depth and precise angle; **(I)** after complete operative procedure, the image showing reposition of the dorsal spinal nerve roots (asterisks), area of SCI (arrow) in more rostral position, and lesioned spinal cord segments (dotted shape) in more caudal position. DREZ, dorsal root entry zone; DREZL, dorsal root entry zone lesioning; SCI, spinal cord injury

**Fig. 3:**
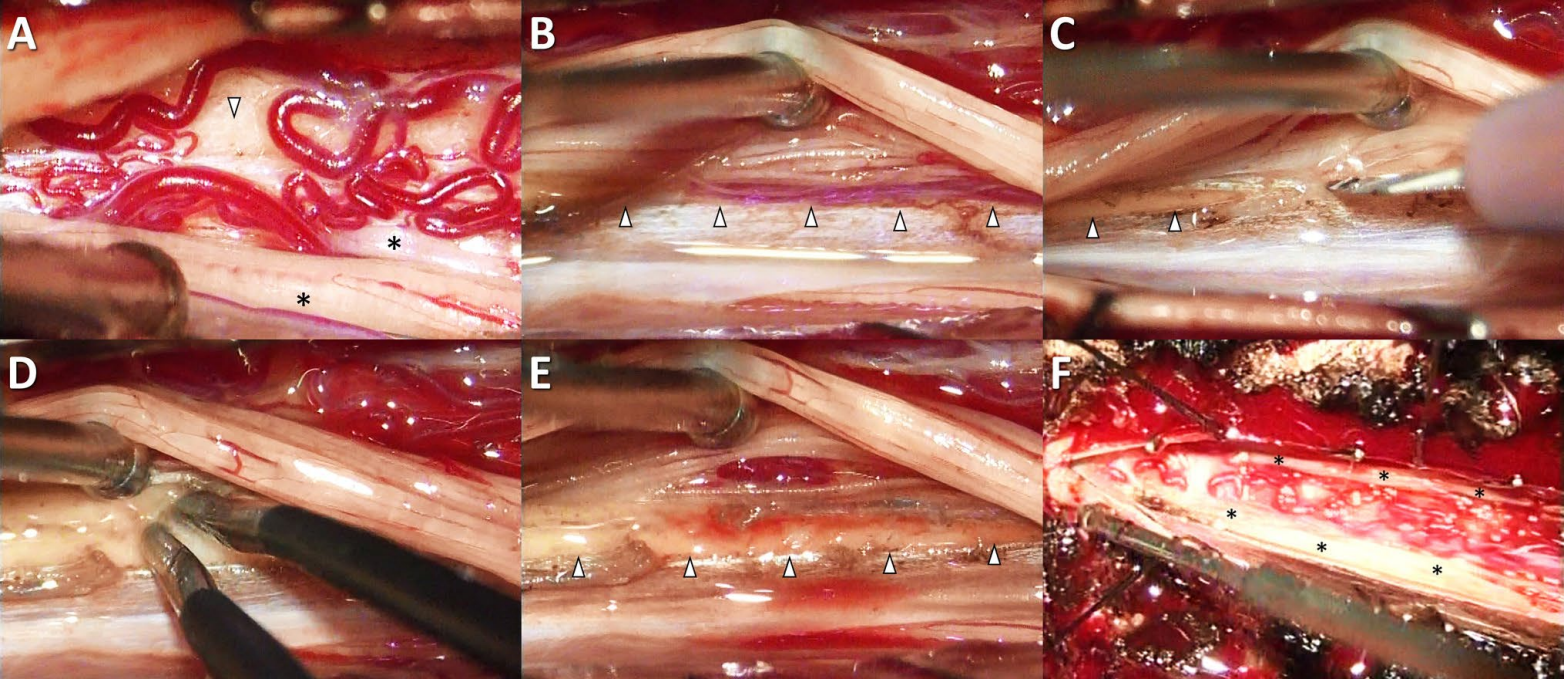
Intraoperative findings and DREZL procedure in a CEI patient with neuropathic pain of the lower limbs. The injury occurred at the level of the cauda equina while the targeted spinal cord segments for DREZL located more rostral to the injury level were structurally normal. The intact spinal cord segments facilitate an effective lesioning procedure. **(A)** exposure of the intact spinal cord (arrowhead) and proximal spinal nerve roots (asterisks) near the spinal cord; **(B)** reflection of the dorsal spinal nerve root to reveal the DREZ (arrowheads); **(C)** incision along the DREZ using an ophthalmic microsurgical blade; **(D)** microsurgical coagulation of the DREZ using fine-tip bipolar coagulation forceps; **(E)** exposure of the dorsolateral sulcus (arrowheads) of the targeted spinal cord segments after the lesioning; **(F)** reposition of the dorsal spinal nerve roots following complete DREZL. CEI, cauda equina injury; DREZ, dorsal root entry zone; DREZL, dorsal root entry zone lesioning

In regards to the findings of this study, the possible explanations of the superior efficacy of DREZL in CEI compared to SCI, or the better outcome of DREZL in border zone pain compared to diffuse pain, can be discussed. Diffuse pain is often caused by extensive spinal cord damage and involvement of multiple neural pathways. These insults make this type of pain more complex to treat. On the other hand, border zone pain usually occurs as a result of nerve root injury, involving a more straightforward and simpler pain mechanism. Additionally, SCI may lead to gliotic changes and scar formation of the spinal cord, and adhesions of the surrounding spinal meninges **(Fig. **2**)**. These changes can complicate intraoperative identification of the dorsolateral sulcus and may hinder effective lesioning, while the spinal cord structure in CEI patients is generally intact and tends to be easier for performing the lesions **(Fig. **3**)**.

Recurrent neuropathic pain can occasionally occur after DREZL. Our study revealed that recurrent pain was more common in the SCI group. The efficacy of DREZL may decrease over time, with incomplete lesioning and new deafferentation pain playing major roles in the development of recurrent pain [88]. With respect to operative complications, DREZL should be performed cautiously in patients with significant residual motor or sensory function, as the operation may cause further impairment [82, 88, 90, 91, 95, 96, 98, 101, 111, 112, 114]. Careful lesioning and intraoperative neuromonitoring are useful for preventing these complications [115].

**Table **4 presents a literature review on the use of DREZL for treating neuropathic pain caused by SCI and CEI [40–42, 44, 82–103, 110–112, 114–123]. **Table **5 lists good and poor predictors of surgical outcomes, operative techniques for improving pain relief, and recommendations, all of which are based on the literature and our results [38, 40–44, 81, 85–87, 90–99, 101–103, 111, 112, 114, 115, 120, 124].

**Table 4:** Literature review of the dorsal root entry zone lesioning procedure for treating pain in spinal cord and cauda equina injuries [40–42, 44, 82–103, 110–112, 114–123]

Authors (year) [reference]	Number of patients with SCI or CEI (*n*)	Injured vertebral levels or neural structures	DREZL technique	Surgical outcomes	Postoperative complications/ adverse outcomes	Comments
Study design	Age (y)	Pain distribution (PD)	Spinal cord (SC) segments of DREZL			
Outcome measurement	Follow-up (F/U, mo)					
Nashold and Ostdahl (1979) [116] Retrospective Percentage of subjective pain relief	*n* = 1, total *N* = 21 Age 35 y F/U > 12 mo	L2-3 PD: Bil legs	RF Low SC and CM	Complete pain relief	None	In addition to brachial plexus avulsion injury, patients with extremity pain from other causes (including SCI) had pain relief by DREZL
Nashold and Bullitt (1981) [82] Retrospective Subjective pain relief	*n* = 13 Age: mean 45.8 y (35–60) F/U: mean 15.9 mo (5–38)	CM or CE 10/13, mid to high T level 3/13 PD - Diffuse 13/13, end-zone 0/13 - Bil 10/13, Unil 2/13, NAD 1/13	RF From one or two SC segments above the level of SCI to the level of injury itself or slight below	Pain relief - Excellent (100%): 7/13 (53.8%) - Good (≥ 50% without necessity of narcotics): 4/13 (30.8%) - Poor (no significant pain relief): 2/13 (15.4%)	- 1 lost hip motion and bladder control without recovery at 17 mo postoperatively - 1 who was able to ambulate with the aid of walking cane had paraplegia after DREZL - 1 who underwent aspiration of cervical syrinx lost effective motor control of the Lt hand	- Failed treatments before DREZL in different patients included phenol injection, decompressive laminectomy, sympathectomy, PR, SCS, cordotomy, cordectomy, aspiration of cervical syrinx - DREZL can be used for central pain associated with spinal trauma and paraplegia
Richter and Seitz (1984) [117] Case series Subjective pain relief	*n* = 10 Age: NAD F/U: mean 5–30 mo	C level 8/10, T level 2/10 PD: NAD	RF C level 8/10, T level 2/10	Early results (during admission for DREZL) - All of patients with C and T SCI had 75% pain relief, 50% of these patients had pain free (100% pain relief) - In 2 treated with T DREZL, 1 had 50% pain relief, the other 1 had no pain relief Late results - 4 treated with C DREZL had good pain relief (> 75%)	Of 8 treated with C DREZL - 2 died - 2 had recurrent pain - 4 had good results in long-term	- Central pain relief in SCI patients can be expected after DREZL
Samii and Moringlane (1984) [83] Retrospective Subjective pain relief	*n* = 5, total *N* = 35 Age: NAD F/U: NAD	Injured level: NAD PD: NAD	RF DREZL segment: NAD	Pain relief - Very Good (70–100%): 2/5 (40%) - Good (50–70%): 2/5 (40%) - Fair (< 50%): 1/5 (20%)	NSD	- DREZL can be used for pain relief from various causes, including injury to the spine and SCI - The overall pain reliefs were satisfactory and long-lasting
Wiegand and Winkelmuller (1985) [84] Retrospective Subjective pain relief	*n* = 15, total *N* = 35 Age: mean 46 y F/U: NSD	Injured level: NAD PD: NSD	RF DREZL segment: NSD	Complete pain relief: 8/15 (53.3%)	None	This study showed best efficacy of DREZL in SCI, cervical root avulsion, and BPI, less efficacy in stump pain and PLP, and failure in sciatic nerve lesion and arachnopathy
Friedman and Nashold et al. (1986) [111], Friedman and Bullitt (1988) [112] Retrospective Pain relief criteria, indicated by medication use and interference with daily activities - Good: complete pain relief or if the pain did not require analgesics or did not interfere with daily activities - Poor: if the patient had residual pain which interfered with normal activities or required the use of narcotic analgesics	*n* = 56 Age 27–72 y F/U 6–72 mo	Injured level: NAD PD - At level of SCI and caudal to level of SCI 31/56, diffuse distal 25/56 - Bil 46/56, Unil 10/56 - Sym in most cases, Asym in few cases	RF DREZL segment: NAD	Pain relief - Overall: good 28/56 (50%), poor 23/56 (41.1%) - Pain at level of SCI and caudal to level of SCI: good 23/31 (74.2%), poor 6/31 (19.4%) - Diffuse distal pain: good 5/25 (20%), poor 17/25 (68%) - Pain with significant burning component: good 10/26 (38.5%), poor 13/26 (50%) - SCI due to closed injury: good 18/36 (50%), poor 14/36 (39%) - Gunshot SCI: good 10/18 (55.6%), poor 6/18 (33.3%) - Unil pain: good 9/10 (90%), poor 1/10 (10%)	- 9 had CSF leak - 1 had epidural hematoma - 2 had weakness and functional deficit - 2 had transient urinary incontinence - 2 had new dysesthesias	- Failed treatments before DREZL in different patients included analgesics, rhizotomy, sympathectomy, cordotomy, cingulotomy, TENS, and SCS - Surgery for end-zone pain in the proximal spinal cord stump is sometimes effective; but it has not been successful for relief of diffuse or sacral burning pain - DREZL maintains pain relief for a longer period of time than does anterolateral cordotomy - Patients who were found to have nerve root avulsions intraoperatively had good prognosis for pain relief
Moossy et al. (1987) [85] Case series Subjective pain relief	*n* = 6 Age: mean 33.8 ± 12.2 y F/U: mean 23 mo	Avulsion injury of L1-S4 spinal nerve roots PD - LE 6/6 - Bil 1/6, Uni 5/6 - Sym 1/6, Asym 5/6	RF L1-S3 SC segments via T11-L2 laminectomy	Complete pain relief: 4/6 (66.7%) (Case 2, 3, 4, 5)	- 2 (Case 1 and 6) had recurrent pain after DREZL - 1 (Case 1) of 2 with recurrent pain underwent second DREZL and had postoperative complete pain relief	- In patients with CM root avulsion, the area of pain was usually less extensive than the neurological deficit - DREZL yields good result in the treatment of pain following CM root avulsion
Moossy and Nashold (1988) [86] Case series Subjective pain relief	*n* = 8 Age: Mean 34.5 ± 10.5 y F/U: NAD	Avulsion injury of T12-S4 spinal nerve roots PD: NAD	RF T11-S4 SC segments via T11-L2 laminectomy	Complete pain relief: 6/8 (75%) (Case 2, 3, 4, 5, 7, 8)	- In 2 (Case 1 and 6) with recurrent pain after DREZL, 1 (Case 1) underwent second DREZL and had postoperative complete pain relief, and 1 (Case 6) had poor result	This series demonstrates the effectiveness of DREZL for the deafferentation pain syndrome caused by CM root avulsion
Powers et al. (1988) [110] Prospective Pain relief criteria, indicated by pain reduction and medication use	*n* = 11, total *N* = 40 Age: NSD F/U: NSD	SCI 9/11, CEI 2/11 PD - Primary truncal (girdle-like) pain of chest and/or abdomen 4/11 - Diffuse pain of LE 8/11 - Midline pain in perineal and scrotal areas 3/11	Laser (CO_2_, argon, Nd: YAG) DREZL segment: NAD	Pain relief regarding injured neural structures - SCI: good 5/9 (55.6%), failure 4/9 (44.4%) - CEI: good 0/2 (0%), failure 2/2 (100%) Pain relief regarding PD - Primary truncal (girdle-like) pain of chest and/or abdomen 4/4 (100%) - Diffuse pain of LE 2/8 (25%) - Midline pain in perineal and scrotal 0/3 (0%)	- 1 had CSF leak and wound dehiscence - 1 CEI patient with recurrent pain underwent reoperation with subsequent failure	This study showed effective pain relief of DREZL in SCI patients, but not in CEI patients
Nashold et al. (1990) [87] Retrospective Pain relief criteria, indicated by medication use and interference with daily activities - Good: no analgesics, no limitation of activity by pain - Fair: no narcotics, no limitation of activity by pain - Poor: narcotics required and/or activity limited by pain	*n* = 18 Age: Mean 39.9 ± 8.2 y F/U: mean 42 mo	C level 1/18, T level 16/18, L level 1/18 PD: NAD	RF with evacuation of spinal cord cysts DREZL was performed into the area of SC damage as well as three cord segments above the lesion. A Unil or Bil DREZL was performed depending on the distribution of pain	Pain relief - Good: 14/18 (77.8%) - Fair: 4/18 (22.2%) - Poor: 0/18 (0%)	- In 3 who underwent second DREZL, 2/3 had good pain relief, and 1/3 had fair pain relief	- Failed treatments before DREZL in different patients included medical treatment, acupuncture, pain interventions, physical therapy, biofeedback, TENS, laminectomy, spinal fusion, myelotomy, SCS, and DBS - The delayed onset of pain following SCI with or without associated progressive neurological deficits may herald the presence of spinal cord cyst - Surgical evacuation of the cyst alone may not relieve pain in the long-term - The combination of cyst evacuation and DREZL is recommended
Young (1990) [40] Retrospective Self-report pain relief	*n* = 20, total *N* = 78 Age: NAD F/U 36 mo	Injured level: NAD PD: NAD	RF vs. CO_2_ laser Bil lesioning from one to two spinal levels rostral to the injured level, and continued caudally to the injured level	Satisfactory outcome (≥ 50% pain relief - Group 1 (lesioning by RF using a 0.5 × 2-mm stainless steel electrode with control of electrical current and duration): SCI 3/5 (60%), CEI 2/2 (100%) - Group 2 (lesioning by CO_2_ laser): SCI 3/6 (50%), CEI 1/2 (50%) - Group 3 (lesioning by RF using a 0.25 × 2-mm stainless steel electrode with control of electrode temperature and duration): SCI 5/9 (55.6%), CEI 2/2 (100%)	NSD	- CEI had better pain relief (83% of patients) than SCI (55% of patients) - RF lesioning with temperature and duration control may be most suitable, reliable, and easiest - Drawbacks of CO_2_ laser included misdirect lesions caused by movement of spinal cord, accidental injury of blood vessel, and unexpectedly large lesions
Kumagai et al. (1992) [88] Retrospective Objective evaluation score of pain relief and subjective pain relief	*n* = 5, total *N* = 15 Age: mean 50.6 y (38–63) F/U 37 mo	Injured level: NAD PD: Bil legs 4/5, Bil thighs 1/5	RF T1-S5 SC segments	Objective pain relief - Excellent (score + 5): 1/5 (20%) - Good (score + 3 to + 4); 2/5 (40%) - Fair (score + 1 to + 2): 1/5 (20%) - Poor (score 0): 1/5 (20%) Subjective pain relief - Initial (2–3 w): 100% in 4/5, 70% in 1/5 - Follow-up: 80% in 1/5, 30% in 3/5, 0% in 1/5	- 1 had sensory loss - 2 had motor weakness - 1 died - 4 had new pain - 1 had transient involuntary leg movement	- Before DREZL, 4/5 underwent SCS with partial pain relief - During DREZL, 4/5 underwent additional modified spinal commissural myelotomy (25–50 mm in length) at the rostral limit of DREZL - Effect of DREZL was decreased over time, the major reasons included incomplete lesioning and new development of deafferentation pain caused by DREZL
Edgar et al. (1993) [89] Retrospective Self-report pain relief	*n* = 46, total *N* = 112 Age: NAD F/U: NSD	Injured level: NAD PD: NAD	Computer-assisted RF DREZL level: NAD	Pain relief - Complete pain relief: 84% of patients - ≥ 50% pain relief: 92% of patients	NSD	- Computer-assisted DREZL was helpful for identifying areas of abnormal focal hyperactivity in the DREZ area, and rendered satisfactory outcomes in intractable posttraumatic spinal deafferentation pain and lumbosacral nerve root avulsion pain
Sampson et al. (1995) [90] Retrospective Pain relief criteria, indicated by medication use and interference with daily activities - Good: complete pain relief, no analgesics - Fair: significant pain reduction, but still required nonnarcotic drugs, pain did not interfere with activities of daily living - Poor: other results	*n* = 39 Age: median 29 y (17–66) F/U: median 12.8 mo (0.25–154.8)	T12-L1 29/39, L2-S1: 10/39 PD: diffuse 16/39, end zone 23/39	RF T10-L2 SC segments	Pain relief - Good: 21/39 (53.8%) - Fair: 8/39 (20.5%) - Poor: 10/39 (25.7%)	T12-L1 injury (*n* = 29) - 4 had permanent weakness - 1 had transient weakness - 2 had CSF leak - 2 had wound infection - 2 had paresthesia - Overall complications 21/29 (72.4%) L2-S1 injury (*n* = 10) - 1 had transient weakness - 1 had UTI - Overall complications 2/10 (20%)	- Higher proportion of good outcome was found in female, blunt injury mechanism, L2-S2 spinal levels, incomplete neurological deficit, longer duration of pain onset, end zone pain, and Unil pain - In most patients, DREZL at the CM can significantly relieve intractable pain
Rath et al. (1996) [91] Retrospective Subjective pain relief: the postoperative pain reduction in percentage of the preoperative level (100%) - Good: pain relief ≥ 75% - Fair: pain relief 25 to < 75% - Poor: pain relief < 25%	*n* = 22, total *N* = 51 Age: mean 47 y (17–74) F/U: mean 54 mo (10–157)	T level 20/22, L level 2/22 PD: end zone 16/22, diffuse 6/22	RF From the level of SCI and extended cephalad for two root segments	Overall pain relief - Good: 11/22 (50%) - Fair: 1/22 (4.5%) - Poor: 9/22 (41%) - Recurrent: 1/22 (4.5%) Patients with end zone pain - Good: 10/16 (62.5%) - Fair: 1/16 (6.25%) - Poor: 4/16 (25%) - Recurrent: 1/16 (6.25%) Patients with diffuse pain - Good: 1/6 (16.7%) - Fair: 0/6 (0%) - Poor: 5/6 (83.3%) - Recurrent: 0/6 (0%)	- 2 had new paresthesia - 1 had recurrent pain - 3 underwent reoperation of DREZL due to unsatisfactory or brief pain relief, good pain relief was achieved in 3/3 after the reoperation - Poor results were seen in 5/7 with syringomyelia	- Persistent good results were found in 12/22 (54.5%) - Most satisfactory results were found in patients with Unil and end zone pain - Diffuse pain poorly responded to DREZL
Rath et al. (1997) [92] Retrospective Subjective pain relief - Good: pain relief ≥ 75% - Fair: pain relief 25 to < 75% - Poor: pain relief < 25%	*n* = 23 Age: mean 47 y (17–74) F/U: mean 58 mo (10–157)	T level 21/23, L level 2/23 PD: end zone 17/23, diffuse 6/23	RF DREZL segment: NAD	Overall pain relief - Good: 11/23 (47.8%) - Fair: 2/23 (8.7%) - Poor: 9/23 (39.1%) - Recurrent: 1/23 (4.4%) Patients with end zone pain - Good: 11/17 (64.7%) - Fair: 1/17 (5.9%) - Poor: 4/17 (23.5%) - Recurrent: 1/17 (5.9%) Patients with diffuse pain - Good: 0/6 (0%) - Fair: 5/6 (83.3%) - Poor: 1/6 (16.7%) - Recurrent: 0/6 (0%)	- 2 had minor complications - In 4 who underwent reoperation of DREZL due to unsatisfactory result, 3/4 had good pain relief, 1/4 had unchanged pain - 5/7 with syringomyelia had poor result - In 5 who underwent combined DREZL with evacuation and drainage of spinal cord cyst, only 1/5 had satisfactory pain relief	In terms of pain relief, end-zone pain had better outcome than diffuse pain
Spaić et al. (1999) [93] Retrospective Self-report pain relief	*n* = 6 Age: mean 25–35 y F/U: mean 9.2 mo (7–12)	T10-L1 with gunshot SCI 6/6 PD - Localized: 5/6, diffuse: 1/6 - Unil: 4/6, Bil: 2/6	MDL At the level of SCI and SC segments corresponding to pain topography	Pain relief - Complete (100%) pain relief: 4/6 (66.7%) - 80% pain relief: 2/6 (33.3%)	None	- Failed treatments before DREZL in different patients included vascularized omental graft implantation and analgesic medications - DREZL was a treatment modality of pain after SCI and should be done before psychological deterioration and narcotic addiction
Sindou et al. (2001) [44] Retrospective VAS - Good: pain relief > 75% - Fair: pain relief 25 to 75% - Poor: pain relief < 25%	*n* = 44 Age NAD F/U: mean 71 mo (12–240)	C: 3/44, T: 22/44, TL: 5/44, L: 14/44 PD: segmental 37/44, infralesional 7/44	MDL Corresponding thoracic cord segments – S4	10-day pain reduction (*n* = 44) - Good: 31/44 (70.5%) - Fair: 8/44 (18.2%) - Poor: 5/44 (11.3%) 3-mo pain reduction (*n* = 44) - Good: 29/44 (65.9%) - Fair: 9/44 (20.5%) - Poor: 6/44 (13.6%) Long-term (> 1 y) pain relief (*n* = 30) - Good: 18/30 (60%) - Fair: 6/30 (20%) - Poor: 6/30 (20%) In long-term follow-up, good pain relief was achieved in 17/25 (68%) patients with segmental pain, while 0/5 (0%) patients with infralesional pain had no benefit from surgery CMI group had better long-term results (82%) than the other groups	- 3 had CSF leak, 1/3 required dural repair - 2 had wound infection - 1 had subcutaneous hematoma that required surgical drainage - 1 had bacteremia due to UTI	- Failed treatments before DREZL in different patients included narcotic analgesics, TCA, AED, TES, SCS, and PR - Patients with segmental pain, CMI or paroxysmal pain alone had better results - DREZL should be reserved for use in patients with predominantly segmental pain - The operation should be performed in the corresponding injured cord segments and in the adjacent one
Prestor (2001) [94] Retrospective VAS	*n* = 7, total *N* = 40 Age: mean 46.6 y (37–57) F/U: mean 49.4 mo (6–84)	SCI: 1/7, syringomyelia: 6/7 PD: NAD	RF Pain-producing SC segments (SCI: T8, syringomyelia: C7-T2)	Pain relief SCI - Poor (no pain relief): 1/1 (100%) Syringomyelia - Excellent (≥ 70%): 5/6 (83.3%) - Good (50–70%): 1/6 (16.7%)	NSD	- Most patients with pain caused by syringomyelia had excellent pain relief
Falci et al. (2002) [95] Retrospective VAS Verbal scale	*n* = 41 Age: median 46 y (19–72) F/U 12–84 mo	T level 34/41, L level 7/41 PD - At-level pain 8/41 - Below-level pain 31/41 - Combined at-level and below-level pain 2/41	MDL Group 1 (*n* = 9): DREZL guided solely by DREZ-induced spontaneous neuroelectrical hyperactivity Group 2 (*n* = 32): DREZL guided by DREZ-induced spontaneous neuroelectrical hyperactivity and transcutaneous C-fiber stimulation of the DREZ	Group 1 - 100% pain relief: 5/9 (55.6%) - 50–100% pain relief: 7/9 (77.8%) - Pain relief < 50%: 2/9 (22.2%) Group 2 - 100% pain relief: 27/32 (84.4%) - 50–100% pain relief: 28/32 (87.5%) In group 2, 26/32 had below-level pain - 100% pain relief: 21/26 (80.8%) - 50–100% pain relief: 22/26 (84.6%)	Partial or complete loss of pinprick sensation in the corresponding dermatomes of DREZL: 22/33 Partial or complete loss of light touch sensation in the corresponding dermatomes of DREZL: 22/33 Motor deficit: 5/35 Other complications - CSF leakage 9.3% - Wound infection 2.3% - delayed spinal instability 7.6% - Pulmonary embolus 2.3% - Wound dehiscence 2.3% Minor pain in new level of sensation - Temporary 2.3% - Permanent 4.7%	- Ineffective preoperative treatment included extensive medical treatment, TCA, AED, baclofen, clonazepam, narcotic analgesics, ITP (narcotic agents, baclofen, clonidine, local anesthetic), and SCS - Intramedullary electrical guidance of DREZL substantially improved pain outcomes in SCI patients compared with conventional techniques
Lee et al. (2002) [115] Case report VAS	*n* = 1 Age 52 y F/U 24 mo	T level PD: Bil T10 dermatomes, Rt T11 dermatome, and Bil LE	MDL DREZL through complete T9-11 laminectomy and Rt T12 laminectomy	Preoperative VAS 9.2 for severe intermittent pain and VAS 5 for less severe pain Postoperative VAS 0 for Bil segmental pain and Rt leg pain; however, VAS temporarily increased to 8.5 for Lt leg pain and then decreased to 0.2 after medical treatment	None	- Failed treatment before DREZL included NSAIDs, carbamazepine, gabapentin, TCA, nonnarcotic analgesics, chiropractic therapy, massage - Intraoperative electrical stimulation and neuromonitoring were used for preventing complications
Spaić et al. (2002) [96] Retrospective VAS	*n* = 26 Age: mean 39 y (24–66) F/U: mean 37 mo (13–50)	T9-L4 PD: confined 22/26, diffuse 4/26	MDL In cases with localized pain, DREZL was performed on the SC segments corresponding to the territory of the pain In cases with diffuse pain below level of spinal cord lesion, DREZL was performed from the cord segment above of the injury site along to the S2 cord segments bilaterally	Initial results - 100% pain relief: 21/26 (80.8%) - 50% pain relief: 1/26 (3.8%) - 80% pain relief: 1/26 (3.8%) - Failure in pain relief: 3/26 (11.6%) Long-term results - 100% pain relief: 14/26 (53.8%) - < 20–90% pain relief: 9/26 (34.6%) - Failure in pain relief: 3/26 (11.6%) Good pain relief was found in patients with intermittent, mechanical, and confined pain	- 7 had recurrent pain during follow-up - 1 had CSF fistula - 1 had wound dehiscence - 2 had anesthesia 2 levels above - 2 had hypoesthesia 2 levels above	- Failed treatments before DREZL in different patients included PR, ITP (morphine), and SCS - Good predictors of surgical outcome after DREZL included confined pain distribution, intermittent rhythm, and mechanical neuropathic pain - Thermal neuropathic pain (burning, boiling, baking, warm) with diffuse infralesional distribution and steady rhythm was the most common resistant to DREZL
Denkers et al. (2002) [118] Systematic review from 13 studies Outcome assessment: NAD	*n* = 248 Age: 7–74 y in 8 studies, NAD in the remaining 5 studies F/U 2–157 mo	Injury level in 7 studies - T level 33–100% - TL level 56–100% - L level 11–19% NAD in 6 studies PD: NAD	RF 11 studies MDL 1 study Laser 1 study DREZL segment: NAD	Good (≥ 50%) to excellent (100%) pain relief: 47.8–100% of all patients	Complications in 7 studies - Weakness 15 - Lost effective hand motor function 1 - Dysesthesia 3 - Loss of proprioception 7 - Transient urinary incontinence 2 - Wound infection 2 - Sexual or bladder - dysfunction 3 - Loss of sphincter control 1 - Delayed myelopathy 1 - Increased leg spasticity 1 - CSF leak 12 - Epidural hematoma 1 - UTI 2 NAD in 3 studies NSD in 3 studies	- The literature supported DREZL as an effective treatment for chronic neuropathic pain in selected patients with SCI - However, the strength of the evidence was weak because of methodologic shortcomings
Lim et al. (2004) [97] Retrospective VAS - Good: pain relief > 75% - Fair: pain relief 25 to 75% - Poor: pain relief < 25%	*n* = 12 (13 operations) Age: mean 49.5 ± 10.6 y F/U: NAD	T level 5/13, TL level 3/13, L level 5/13 PD - T10-S3 dermatomes - Unil 1/13, Bil 10/13, NAD 2/13 - Sym 10/13, Asym 1/13, NAD 2/13 - Diffuse 2/13, border zone 11/13	RF T8-S3 SC segments	Pain relief - Good: 8/13 (61.5%) - Fair: 4/13 (30.8%) - Poor: 1/13 (7.7%) Treatment success - Mechanical pain group: 8/10 (80%) - Intermittent pain group: 6/7 (85.7%) - Continuous pain group: 2/6 (33.3%) - Localized pain group: 7/11 (63.6%) Poor outcome - Diffuse pain: 1/2 (50%) Treatment failure - Thermal pain group: 2/2 (100%)	- Complication: NAD - 1 underwent second DREZL for spastic bladder pain	- RF DREZL is more effective in intermittent and/or mechanical pain than continuous and/or thermal pain
Spaić et al. (2005) [98] Retrospective VAS	*n* = 38 Age: mean 36 y (22–48) F/U: NAD	T level 2/38, CM 36/38 PD: NAD	Group A: standard MDL 24/38 Group B: modified MDL with dorsal horn suction technique 14/38 In cases with localized pain topography, DREZL was performed on SC segments corresponding to pain territory In cases with diffuse pain below level of SC lesion, DREZL was performed from the one SC level above the injury site along to the S1 cord segment bilaterally	Long-term pain relief Group A - Pain relief > 50%: 77% of patients - Complete pain relief: 50% of patients Group B: - Pain relief > 50%: 85% of patients - Complete pain relief: 64% of patients	- 4 had CSF leak - 2 had wound dehiscence - 4 with incomplete SCI had sensory loss in dermatomes corresponding to the operated cord segments (2/4 in group A, and the other 2/4 in group B) - 2 had worsening of motor function (2/2 in group A)	- Completeness of DREZL might play major role in providing better long-term result after surgery - Intermittent rhythmic and defined Bil or Unil pain territory were the most important features successfully cured by DREZL
Ruiz-Juretschke et al. (2011) [119] Retrospective VAS	*n* = 2, total *N* = 18 Age - Case 17: 77 y - Case 18: 60 y F/U: 28 mo (6–108)	Injury level - Case 17: T9-11 - Case 18: T10-12 PD: NAD	RF DREZL Segment - Case 17: Bil T10-12 - Case 18: Bil T10-L1	Pain relief on discharge - Case 17: 100% (excellent) - Case 18: 40% (moderate) Pain relief on final follow-up - Case 17: NAD - Case 18: 20% (poor)	NSD	- Failed treatments before DREZL in different patients included medical therapies, SCS, ITP (morphine), and thalamic DBS - DREZL through RF was indicated in segmental pain secondary to SCI
Chun et al. (2011) [99] Retrospective VAS - Good: pain relief > 75% - Fair: pain relief 25 to 75% - Poor: pain relief < 25%	*n* = 38 Age: mean 49 y (32–69) F/U: mean 42 mo (19–84)	T level 5/38, CM 33/38 PD: diffuse 15/38, segmental 23/38	MDL In diffuse, thermal or continuous pain, DREZL was performed bilaterally on the injured and scarred SC segments in the lump and irritative zones	Pain relief - Good: 30/38 (78.9%) - Fair: 4/38 (10.5%) - Poor: 4/38 (10.5%) Mean VAS - Preoperative 8.58 - Immediate postoperative 3.05 - 6-mo postoperative 2.52 - Final follow-up 2.16 Good pain relief - Segmental 19/23 (82.6%) - Diffuse 11/15 (73.3%)	- 1 had CSF leak	- Manipulation and cutting of all injured rootlets with adhesion, avulsion, and scar formation, regardless of pain distribution, may improve the surgical outcomes of DREZL
Awad et al. (2013) [100] Retrospective VAS - Excellent: complete pain relief - Good: pain relief > 50% - Mild: pain relief < 25% - Poor: no pain relief or pain exacerbation	*n* = 6 Age: mean 54.5 y (38–66) F/U: mean 58.8 mo (1.2–144)	T level 6/6 PD: end zone 6/6	RF DREZL: NAD	Pain relief - Excellent: 2/6 (33.3%) - Good: 3/6 (50%) - Mild: 1/6 (16.7%) - Poor: 0/6 (0%)	None	- Most patients reported improvement in quality of life (general activity, mood, sleeping, and enjoyment of life) after DREZL
Mehta et al. (2013) [120] Systematic review from 11 studies Outcome assessment - VAS 4/11 studies - Self-report pain relief 3/11 studies - Pain relief, as indicated by subsequent treatment and activity levels 4/11 studies	*n* = 290 Age 17–74 y F/U: NAD	Injury level - C level 11/290 - T level 75/290 - CM 108/290 - L level 7/290 - CE 24/290 - NSD 65/290 PD: NAD	MDL 6/11 studies RF 5/11 studies DREZL segment: NAD	Good pain relief - C level 67% of patients - T level 0–60% of patients - CM 52–100% of patients - L level 88% of patients - CE 25–88% of patients	NAD	- Most studies showed efficacy of DREZL for reduction of segmental pain or pain from CMI or CEI
Tao et al. (2014) [101] Retrospective VAS - Excellent: pain relief > 75% - Good: pain relief 25 to 75% - Poor: pain relief < 25%	*n* = 35 Age: mean 50.6 y (28–72) F/U: 36.1 mo (12–72)	T level 11/35, L level 24/35 PD - At-lesion-level pain 33/35 - Below-lesion-level pain 1/35 - Above-lesion-level pain 1/35	MDL The extension of DREZ coagulation segments depended on the pain territory	Postoperative pain relief At 2 w - Excellent: 33/35 (94.3%) - Poor: 2/35 (5.7%) At 3 mo - Excellent: 30/35 (85.7%) - Good: 2/35 (5.7%) - Poor: 3/35 (8.6%) Long-term (*n* = 33 because 2 died) - Excellent: 24/33 (72.7%) - Good: 6/33 (18.2%) - Poor: 5/33 (9.1%)	- 6 had hypoesthesia - 2 had transient muscle weakness - 1 had transient urinary retention - 1 had CSF leak - 1 had wound dehiscence - 2 died (1 and 1 died of pneumonia and suicide, respectively)	- Failed treatments before DREZL in different patients included narcotic analgesics, TCA, AED, PR, SCS, and ITP - Both patients with below- and above-lesion-level pain had poor results - The best candidate for DREZL is patients with at-lesion-level pain
Chivukula et al. (2015) [121] Retrospective VAS	*n* = 11, total *N* = 83 Age: NSD F/U - T SCI: mean 195.6 mo (36–279.6) - L SCI: mean 147.6 mo (26.4–260.4)	T level 8/11 L level 3/11 PD - T SCI: Bil 8/8 - L SCI: Unil 3/3	RF DREZL extending from just caudal to the most caudal normal dorsal rootlets to just caudad to the most rostral normal dorsal rootlets	T SCI - Mean preop VAS 8 - Mean postop VAS 3.5 - Mean pain reduction 55.3% (42.9–70%) - Years of satisfactory pain relief: <1 y in 1/8 (12.5%), 1–5 y in 3/8 (37.5%), > 5 y in 4/8 (50%) L SCI - Mean preop VAS 8.3 - Mean postop VAS 3.3 - Mean pain reduction 58.9% (50–66.7%) - Years of satisfactory pain relief: <1 y in 0/3 (0%), 1–5 y in 2/3 (66.7%), > 5 y in 1/3 (33.3%)	None	- Failed operative treatments before DREZL in different patients - T SCI: SCS, limb amputation, spinal structural surgery, ITP - L SCI: none
Urasaki et al. (2016) [114] Case report VAS	*n* = 1 Age 71 y F/U 4 mo	T12 PD - Bil LE, Bil inguinal and gluteal regions, perineum, genitalia, anus - Sym - Diffuse: Bil LE, perineum, genitalia, anus - Segmental: Bi inguinal and hip regions	MDL Combined Bil extensive DREZL from T8-T12 SC segments, Bil dorsal horn aspiration, and dorsal root resection from L1-S1	- Complete pain relief of segmental pain in Bil inguinal and gluteal regions - Partial pain relief of diffuse pain in Bil LE and genitalia - No improvement of diffuse pain in perineum and anus	- Partially impaired sensation extended to the upper abdomen (T8 dermatome) - Completely impaired sensation at Bil inguinal regions - New segmental neuropathic pain with allodynia in the abdomen at 4 mo after the surgery	- Extensive DREZL combined with dorsal horn aspiration and dorsal root resection, might be effective in treatment of diffuse thermal pain, where conventional DREZL is generally ineffective
Piyawatthanametha et al. (2017) [42] Retrospective VAS	*n* = 6, total *N* = 40 Age: NSD F/U: 18 mo (3–30)	NAD PD: NAD	MDL DREZL segment: NAD	Pain relief - Good (≥ 50% pain relief): 4/6 (66.7%) - Pain free (100% pain relief): 2/6 (33.3%)	- 3 had complications	- Outcome of DREZL for SCI pain and BPI with root avulsion pain was better than that for neuropathic pain caused by PLP, cancer pain, CRPS, and CEI
Falci et al. (2018) [102] Case series Verbal scores of 1–10	*n* = 3 Age - Case 1: 49 y - Case 2: 54 y - Case 3: 45 y F/U - Case 1: 18 mo - Case 2: 30 mo - Case 3: 132 mo	Injury level - Case 1: T5 - Case 2: T4 - Case 3: T3 PD - Below-level pain 3/3 - Case 1: Bil below-level pain from T6 dermatomes - Case 2: Bi l below-level pain from T5 dermatomes - Case 3: Bil below-level pain from T4 dermatome	MDL DREZL caudal to the level of SCI	- Complete pain relief in the gluteal region, rectum, genitalia, upper leg, lower leg, and feet: 3/3 (100%) - Residual burning and electrical sensations in the feet: 1/3 (33.3%) - Residual nonpainful tingling and warmth sensation in the feet: 1/3 (33.3%) - Completely wean off preoperative medications (fentanyl transdermal system, gabapentin, duloxetine): 2/3 (66.7%)	- 1 had postoperative pseudomeningocele, which resolved within 6 mo without surgical treatment	- Failed treatments before DREZL in different patients included narcotic, antidepressant, AED, spinal cord untethering surgery with expansive duraplasty and cyst shunting, and DREZL at and cephalad to the level of SCI - The spinal cord DREZ caudal to the level of SCI can be a primary generator of SCI below-level central pain
Bing et al. (2019) [103] Retrospective VAS - Excellent: > 75% of pain reduction, remarkable improved sleep quality, no analgesics taken - Good: pain reduction > 50%, improved sleep quality, less analgesics - Poor: < 50% of pain reduction, sedative drug assisting with sleep, no decrease in analgesics	*n* = 42 Age: NAD F/U: mean 40 mo (12–71)	T level 3/42, TL level 3/42, L level 36/42 PD - T11-S5 dermatomes - Unil 4/42, Bil 38/42 - Sym 35/42, Asym 7/42	MDL DREZL segment - T9-S5 SC segments - Unil 4/42, Bil 38/42	Pain relief - Excellent or good: 35/42 (83.3%) - 100% pain relief: 21/42 (50%) - 50% pain relief: 14/42 (33.3%) - Less than 50% pain relief: 6/42 (14.3%)	- 18/42 (42.9%) had temporary tingling in the dermatomes corresponding to the upper border of the DREZL segment - 3 had permanent tingling pain; 2/3 underwent additional DREZL in the upper margin of previous DREZL segment with complete pain relief - 2/4 with Unil LE pain had postoperative persistent irritant pain in the contralateral LE; this irritant pain subsided in 1/2 - 1 had wound dehiscence - 1 had UTI	- Nerve root injury pain had a good prognosis after DREZL - Effect of DREZL for SCI segmental pain may be uncertain - DREZL may be ineffective in perineal neuropathic pain caused by CMI
Galafassi et al. (2021) [122] Systematic review from 10 studies with SCI patients Outcome assessment: NAD	*n* = 217 (9 studies) and NSD in 1 study Age: NAD F/U: NAD	Injury level: NAD PD: NAD	RF 7/10 studies MDL 3/10 studies DREZL segment - C level: NSD - T level: NSD - CM: NSD	Overall pain relief 48–87%	NAD	- DREZL is considered as an alternative for the treatment of chronic pain, particularly in patients who do not tolerate adverse effects of medication or who have intractable pain
Mongadi et al. (2021) [123] Systematic review from 15 studies VAS, personal report - Good: pain reduction > 75%, residual VAS score < 3, and no longer need for pain killers - Fair: pain reduction between 75% and 50%, residual VAS < 5, and/or the need for moderate doses of analgesic drugs - Poor: pain reduction < 50%, residual VAS 3–6, with peak rates as higher than 6, and/or the need for high doses of analgesic drugs	*n* = 301 Age: NAD F/U 12–144 mo	Injury level: NAD PD: NAD	RF 10/15 studies MDL 5/15 studies DREZL segment: NAD	Pain relief - Good: 168/301 (55.8%) - Fair: 35/301 (11.6%) - Poor: 82/301 (27.2%) Good long-term pain relief (> 3 y): 92/153 (60.1%)	NSD	- SCI may benefit from DREZL - Good outcome was found in 55.8% of patients and remained substantially stable over time
Du et al. (2023) [41] Retrospective Numeric rating scale (NRS) and global impression of change (GIoC)	*n* = 9, total *N* = 19 Age: NSD F/U: NSD	Injured level - SCI (*n* = 7): C level 2/7, T12-L1 5/7 - CEI (*n* = 2) at L2: 2/2 PD - SCI: Bil 5/7, Unil 2/7 - CEI: Bil 2/2	MDL DREZL segment - SCI: C5-T2, T10-S2 SC segments - CEI: L1-S5 SC segments	Overall pain relief - SCI: 33–67% - CEI: 80–86%	NSD	- Before DREZL, SCS was ineffective for pain relief in all patients - Surgical outcome of DREZL for pain caused by CEI was better than that of SCI
Present study Retrospective study NPRS	*n* = 12 Age: mean 45.2 ± 14.1 y F/U: 45.5 mo (17–104)	SCI 6/12, CEI 6/12 PD SCI group - Diffuse 4/6, border zone 2/6 - Bil 6/6, Unil 0/6 CEI group - Diffuse 0/6, border zone 6/6 - Bil 3/6, Unil 3/6	MDL DREZL segment - SCI group: T7-S3 - CEI group: L1-S2	Median percentage of pain improvement between SCI vs. CEI groups - One-year after DREZL: SCI group 73.9% vs. CEI group 88.9% (*p* = 0.110) - Long-term after DREZL: SCI group 31.1% vs. CEI group 83.4% (*p* = 0.020) Favorable outcome (≥ 70% reduction in NPRS) between SCI vs. CEI groups - One-year after DREZL: SCI group 4/6 (66.7%) vs. CEI group 5/6 (83.3%) (*p* = 0.999) - Long-term after DREZL: SCI group 1/6 (16.7%) vs. CEI group 6/6 (100%) (*p* = 0.015) Median percentage of pain improvement between diffuse vs. border zone pain - One-year after DREZL: diffuse pain 58.9% vs. border zone pain 84.5% (*p* = 0.174) - Long-term after DREZL: diffuse pain 21.1% vs. border zone pain 78.9% (*p* = 0.008) Favorable outcome (≥ 70% reduction in NPRS) between diffuse vs. border zone pain - One-year after DREZL: diffuse pain 2/4 (50%) vs. border zone pain 7/8 (87.5%) (*p* = 0.491) - Long-term after DREZL: diffuse pain 0/4 (0%) vs. border zone pain 7/8 (87.5%) (*p* = 0.010)	Complications - None in SCI - 3/6 in CEI group (2 had transient LE weakness, and 1 had permanent LE weakness) Recurrent pain after DREZL - 2/6 in SCI group had recurrent pain several mo following DREZL, 1/2 with recurrent pain was failed to SCS - None in CEI group	- Surgical outcome of DREZL in patients with neuropathic pain caused by CEI was significantly better than that in patients with neuropathic pain caused by SCI - Recurrent pain after DREZL was more common in SCI patients - Border zone neuropathic pain was a good predictor of pain relief following DREZL - Diffuse neuropathic pain below level of SCI was associated with poorer outcome - Intraoperative spinal nerve root stimulation was useful in localization of precise SC segments for DREZL

AED, antiepileptic drug; Asym, asymmetrical; Bil, bilateral; BPI, brachial plexus injury; C, cervical; CE, cauda equina; CEI, cauda equina injury; CM, conus medullaris; CMI, conus medullaris injury; CRPS, complex regional pain syndrome; CSF, cerebrospinal fluid; DBS, deep brain stimulation; DREZ, dorsal root entry zone; DREZL, dorsal root entry zone lesioning; F/U, follow-up; ITP, intrathecal pump; L, lumbar; LE, lower extremity; Lt, left; MDL, microsurgical dorsal root entry zone lesioning; mo, months; n, number of patients with SCI or CEI in the study; N, total number of patients in the study; NAD, no available data in the study; NSAIDs, non-steroidal anti-inflammatory drugs; NSD, no specific data for patients with spinal cord injury or cauda equina injury in the study; PD, pain distribution; PLP, phantom limb pain; PR, posterior rhizotomy; RF, radiofrequency; Rt, right; S, sacral; SC, spinal cord; SCI, spinal cord injury; SCS, spinal cord stimulation; Sym, symmetrical; T, thoracic; TCA, tricyclic antidepressant; TENS, transcutaneous electrical nerve stimulation; TES, transcutaneous electrical stimulation; Unil, unilateral; UTI, urinary tract infection; VAS, visual analogue scale; w, weeks; y, years

**Table 5:** Predictors of surgical outcomes, strategies to improve pain relief, and recommendations for dorsal root entry zone lesioning in patients with spinal cord and cauda equina injuries [38, 40–44, 81, 85–87, 90–99, 101–103, 111, 112, 114, 115, 120, 124]

	Authors [reference]
*Good predictors of surgical outcome*	
Conus medullaris nerve root avulsion	Moossy et al. [85], Moossy and Nashold [86], Friedman and Nashold [111], Friedman and Bullitt [112]
Conus medullaris injury	Sindou et al. [44], Mehta et al. [120]
Cauda equina injury	Young [40], Du et al. [41], Mehta et al. [120]
Nerve root injury	Bing et al. [103]
Paroxysmal pain	Sindou et al. [44], Spaić et al. [96], Spaić et al. [98], Lee et al. [115]
Border zone pain	Sampson et al. [90], Rath et al. [91], Rath et al. [92], Spaić et al. [96], Spaić et al. [98], Tao et al. [101], Friedman and Nashold [111], Friedman and Bullitt [112], Mehta et al. [120], present study
Associated syringomyelia	Prestor [94]
*Poor predictors of surgical outcome*	
Diffuse pain	Rath et al. [91], Rath et al. [92], Spaić et al. [96], Friedman and Nashold [111], Friedman and Bullitt [112], present study
Below-lesion-level pain and above-lesion-level pain	Lammertse and Falci [81], Spaić et al. [96], Tao et al. [101]
Continuous pain	Spaić et al. [96], Lim et al. [97]
Thermal pain	Spaić et al. [96], Lim et al. [97]
Perineal neuropathic pain	Bing et al. [103]
*Surgical strategies to improve pain relief*	
Completeness of DREZL	Spaić et al. [98]
The combination of posttraumatic SC cyst evacuation and DREZL	Nashold et al. [87]
Inclusion of posttraumatic cystic and noncystic (myelomalacic) lesion into surgical treatment planning	Lammertse and Falci [81]
Utilization of intramedullary electrical guidance of DREZL	Falci et al. [95]
Manipulation and cutting of all injured rootlets with adhesion, avulsion, and scar	Chun et al. [99]
A combination of DREZL with dorsal horn aspiration and dorsal root resection for diffuse thermal pain	Urasaki et al. [114]
DREZ on the spinal cord caudal to the level of SCI for below-level pain	Falci [102]
*Recommendations*	
Performing DREZL before development of psychological problems and narcotic addiction	Spaić et al. [93]
Consideration of DREZL in patients with predominantly border zone pain	Sindou et al. [44], Sindou [124], present study
Performing DREZL in the corresponding injured spinal cord segments and in the adjacent one	Sindou et al. [44], Sindou [124]
Use of intraoperative spinal nerve root stimulation for localization of precise spinal cord segments	Sitthinamsuwan et al. [38], Piyawattanametha et al. [42], Sitthinamsuwan et al. [43], present study
Use of intraoperative electrical stimulation and neuromonitoring for prevention of neurologic complications	Lee et al. [115]

DREZL, dorsal root entry zone lesioning; SC, spinal cord; SCI, spinal cord injury

In a particular case, Patient 3 with T9 vertebral injury had the upper margin of diffuse pain at the bilateral T8 dermatomes which can be defined as “pain above the injury level”. This pattern of neuropathic pain is relatively less common than pain below the injury level, and tends to be associated with SCI or complex regional pain syndrome, whereas border zone pain may originate from damage to spinal nerve roots or spinal cord [47, 50, 63]. In a study of pain patterns in 100 SCI patients by Verma et al, 90 (90%) individuals reported occurrence of pain. Of 90 patients, 23 (25.6%) had pain above the injury level and the remaining 67 (74.4%) had pain below the injury level [64]. Our literature review showed that pain above the injury level poorly responded to DREZL [81, 96, 101]. Patient 3 also had unsatisfactory (40%) postoperative pain relief even though the upper border of lesioning was extended superior to and covered the spinal cord segments corresponding to the painful region.

## Strengths and limitations of the study

A strength of this study is the clear separation of patients with SCI from those with CEI, with no patients having concurrent injuries. To our knowledge, no previous studies have statistically compared the outcomes of DREZL for neuropathic pain between SCI patients and CEI patients. Despite its small sample size, our study achieved statistically significant results in important aspects, such as long-term postoperative pain relief and outcomes between the SCI and CEI groups, as well as between patients with diffuse and border zone pain. However, the small population size is a limitation that may have affected the significance of some analyses. Increasing the number of DREZL cases for SCI and CEI is challenging, as the procedure is less commonly used for these conditions than for brachial plexus avulsion injury pain. Most original publications have small patient populations, although systematic reviews pooling data have recently become more common.

## Conclusions

Although DREZL is a rare operative procedure, it remains an effective option for relieving neuropathic pain following SCI and CEI. It should be considered in refractory patients for whom conventional therapies and neuromodulation have failed. On the basis of our results, the surgical outcomes of patients with CEI were superior to those of patients with SCI. The long-term outcome was most favorable in patients with border zone neuropathic pain, whereas those with diffuse pain below the level of SCI responded poorly to the procedure and had a high recurrence rate.

## Data Availability

No datasets were generated or analysed during the current study.
